# *Toll-like receptor 2 (−196 to −174) del* and *TLR1 743 A > G* gene polymorphism—a possible association with drug-resistant tuberculosis in the north Indian population

**DOI:** 10.3389/fmicb.2023.1305974

**Published:** 2024-01-24

**Authors:** Deepika Varshney, Shoor Vir Singh, Keshar Kunja Mohanty, Santosh Kumar, Nitin Varshney, Ekata Sinha, Sushanta Kumar Barik

**Affiliations:** ^1^ICMR-National JALMA Institute for Leprosy and Other Mycobacterial Diseases, Agra, Uttar Pradesh, India; ^2^Department of Biotechnology, Institute of Applied Sciences & Humanities, GLA University, Mathura, Uttar Pradesh, India; ^3^Department of Chest and Tuberculosis, S. N. Medical College & Hospital, Agra, Uttar Pradesh, India; ^4^Department of Agricultural Statistics and Computer Centre, N. M. College of Agriculture, Navsari Agricultural University, Navsari, Gujarat, India

**Keywords:** Toll-like receptors, drug-resistant tuberculosis, genotyping, TNF-α, IL-6, IFN-γ

## Abstract

**Objectives:**

The objective of this study is to analyze the association between *TLR2 deletion (−196 to −174)* and *TLR1 743 A > G* gene polymorphism with drug resistant tuberculosis (PTB, MDR-TB, and XDR-TB) in a population from Agra, Uttar Pradesh.

**Methods:**

The present case–control study included 101 pulmonary TB patients, 104 multidrug-resistant TB patients, 48 extremely drug-resistant TB patients, and 130 healthy and unrelated controls residing in the same locality. The genotyping method for *TLR2 deletion (−196 to −174)* was carried out by allele-specific polymerase chain reaction (PCR), and *TLR1 743 A > G* gene polymorphism was performed by hybridization probe chemistry in Roche Real-Time PCR. Genotype and allele frequencies were analyzed by the chi-square test. Cytokine levels were measured by ELISA and compared using Mann–Whitney and Kruskal–Wallis tests.

**Results:**

The frequency of heterozygous (*Ins/del*) genotypes for *TLR2 (−196 to −174)* polymorphism was predominant in XDR-TB patients (0.57), whereas heterozygous A/G genotype for *TLR1 743 A > G* single nucleotide polymorphism (SNP) was predominant in healthy controls (0.57) for *TLR1 743 A > G* gene polymorphism. The heterozygous genotype of *TLR2 deletion* polymorphism was found to be significantly higher in XDR-TB (*p* = 0.0001). *TLR1 743 A > G* SNP, AG genotypes were found to be significantly associated with healthy controls than PTB (*p* = 0.047). The level of serum cytokines (IL-6, TNF-α, and IFN-γ) was also found to be significantly different among TB patients and healthy controls.

**Conclusion:**

The findings suggested that in the present population, the heterozygous (*Ins/Del*) genotype and deletion allele of *TLR2 deletion (−196 to −174)* polymorphism are associated with the risk for the development of drug-resistant TB. Furthermore, for *TLR1 743 A > G* gene polymorphism, A/G genotype, and G allele are found associated with healthy controls, suggesting the protective role against TB.

## 1 Introduction

According to epidemiological data provided by the World Health Organization, the global count of newly reported Tuberculosis (TB) cases in 2021 reached 10.6 million cases, reflecting a 4.5% rise from the 10.1 million cases recorded in 2020. Between 2020 and 2021, there is an estimated 3.6% rise in the Tuberculosis (TB) incidence rate. In 2021, an estimated rate of DR-TB was observed to be 450,000 cases, which is reported to have increased 3.1% from 437,000 in 2020 (World Health Organization, 2022). The diverse host immune response to *Mycobacterium tuberculosis* infection is influenced by both host genetic factors and the reaction of the host to the infection (Berrington and Hawn, 2007). The host cell recognizes Mycobacteria through various receptors, and one of them is Toll-like receptor (TLR). TLR recognizes molecules such as lipoprotein and lipopolysaccharides known as PAMPs that are present in bacteria and bind to host pattern recognition receptors (PRRs) (Jahantigh et al., 2013). TLR1 recognizes tri-acylated lipopeptide, and TLR6 recognizes di-acyl lipopeptide (Buwitt-Beckmann et al., 2005; Omueti et al., 2005). TLR1 recognizes 19 kD lipoprotein of *M.tb* (Takeuchi et al., 2002), and TLR2 recognizes 19 kD lipoproteins and lipoarabinomann and other PAMPs molecule of *M.tb* (Brightbill et al., 1999). Dendritic cells, macrophages, natural killer cells, and T and B lymphocytes express TLRs (Vidya et al., 2018). The human *TLR2* gene is situated on chromosome 4q32, and TLR2 molecules consist of two non-coding exons and one coding exon (Haehnel et al., 2002). Toll-like receptors involved in recognizing *M.tb* include TLR1, TLR2, TLR4, TLR6, TLR8, and TLR9 (Thada et al., 2013). The initial defense of our immune system is the innate immune response (Chen et al., 2010). In the previous study by Mittal et al., the frequency of −196 to −174 del polymorphism of the *TLR2* gene was compared between PTB cases and healthy controls in population of Agra (Uttar Pradesh), and the similar allele frequencies were reported in both cases and controls; no significant difference was observed in genotype frequencies of TB patients and healthy controls (Mittal et al., 2018). In addition, the previous study by Sinha et al., where they examined the frequency of the *743 A > G* gene polymorphism in the *TLR1* gene, compared the cases of pulmonary tuberculosis (PTB) with healthy controls from the Agra region. The study revealed that AG genotypes were significantly more prevalent in healthy controls than in PTB cases. Additionally, the research indicated that the heterozygous condition at the *743 A > G* locus conferred a degree of resistance to the disease by restricting bacterial growth (Sinha et al., 2014).

*TLR2 (−196 to −174) del* polymorphism is studied in various populations, suggesting the role in susceptibility toward TB. Khan et al. in their study has reported the higher allele frequency of the deletion allele of *TLR2 (−196 to −174) (Ins/del)* polymorphism in TB-infected patients in the Pakistani population (Khan et al., 2014). The study conducted by Ma et al. revealed an association between alleles encoding the *743 A > G* variant and cases of pulmonary tuberculosis (PTB) in the African-American population (Ma et al., 2007). Uciechowski et al. discovered that the *743A > G* variant was linked to susceptibility to tuberculosis (TB) in a low-endemic country, and at the same time, they did not observe any association in the Ghanaian population (Uciechowski et al., 2011). The role of these polymorphisms is unexplored in drug-resistant tuberculosis. Drug resistance develops due to some possible causes such as (1) drug compliance, (2) bacterial genotype, and (3) host genetic factors (Ben-Kahla and Sahal, 2016). The prevalence of DR-TB is high in India and is listed as one of the 30 high burden countries for DR-TB (World Health Organization, 2022). Observing the prevalence rate and the severity of the disease, it becomes imperative to conduct such studies which look into the cause of the disease.

## 2 Materials and methods

### 2.1 Ethics statement

The study protocol was approved by the Institute Human Ethics Committee and established following the guidelines outlined by the Indian Council of Medical Research (2017).

### 2.2 Study population

The tuberculosis patients were recruited from the Outpatient Department (OPD) of the Department of Chest and Tuberculosis, Sarojini Naidu Medical College, Agra, and ICMR-National JALMA Institute for Leprosy and Other Mycobacterial Diseases (NJIL & OMD), who were registered in the OPD from Monday to Friday from 2019 to 2021. Informed consent was obtained from all participants. Only those patients were included who consented to participate in the study. Most of these patients were inhabitants of Agra or neighboring areas within the state of Uttar Pradesh. The patients were clinically examined by the chest physician and evaluated following the guidelines of TB India (2017). Based on the findings of AFB smear positivity in sputum, CBNAAT, radiography, LPA and DST, the participants were categorized as follows.

#### 2.2.1 Pulmonary tuberculosis (PTB) patients

PTB patients were those who were diagnosed based on smear positivity for AFB, CBNAAT (rifampicin resistance not detected), and clinical manifestations, following the guidelines of NTEP. A new TB patient is defined as someone with pulmonary tuberculosis (PTB) has never undergone treatment for TB or has taken anti-TB drugs for less than 1 month. Patients with seropositive HIV infection or suffering from diabetes or any other immunosuppressive disorders were excluded from the study.

#### 2.2.2 Multi drug-resistant tuberculosis (MDR-TB) patients

Patients classified as MDR are those whose biological specimen demonstrates resistance to both isoniazid and rifampicin, with or without resistance to other first-line anti-TB drugs. Diagnosis of MDR-TB patients was confirmed based on the CBNAAT test (resistant to rifampicin) and FL-LPA, SL-LPA, and LC DST, following the guidelines (Guidelines on Programmatic Management of Drug-Resistant Tuberculosis in India, 2017). Patients who were showing co-infection with HIV or any other immunosuppressive disease were excluded from the study.

#### 2.2.3 Extensively drug-resistant tuberculosis (XDR-TB) patients

XDR patients meet the criteria for MDR/RR-TB and additionally showed resistance to any fluoroquinolone (levofloxacin or moxifloxacin) and at least one additional Group A drug (either bedaquiline or linezolid or both). XDR patients were diagnosed based on FL-LPA, SL-LPA, and LC DST following the guidelines (Guidelines on Programmatic Management of Drug-Resistant Tuberculosis in India, 2017).

#### 2.2.4 Healthy controls

Healthy unrelated individuals from the same socioeconomic background, including those accompanying patients to hospitals and students visiting our institute for training and project work, were enlisted as healthy controls. Schedules containing subject details were filled, and individuals with a recent history of fever, viral infection, other illnesses, or any immunological diseases, as well as those who had received treatment for tuberculosis or leprosy in the past or had a family history of tuberculosis, were excluded from the study. The current study included individuals aged 18–60 years who were in overall good health.

### 2.3 Selection of gene polymorphism

*TLR2 (−196 to −174) del* polymorphism is reported to alter the function of TLR2 promoter and decrease transcription-responsive promoters (Tahara et al., 2008). The *TLR1* gene polymorphism *743 A > G* located in the extracellular domain is associated with ligand-binding activity (Ma et al., 2007). Meta-analysis revealed that *TLR2 (−196 to −174) del* polymorphism and *TLR1 743 A > G* gene polymorphism were significantly associated with TB disease (Varshney et al., 2022). Although there are reports related to frequencies and association between *TLR2 (−196 to −174) del* polymorphism (Mittal et al., 2018) and *TLR1 743 A > G* gene polymorphism (Sinha et al., 2014), from Agra population, no information is available about the host gene polymorphism among the drug-resistant tuberculosis. The present study will be the first report on the frequencies of *TLR2 (−196 to −174) del* and *TLR1 743 A > G* gene polymorphisms in drug-resistant tuberculosis patients. Hence, *TLR2 (−196 to −174) del* polymorphism and *TLR1 743 A > G* gene polymorphism were selected.

### 2.4 Genotyping for the *TLR2* gene

DNA extraction was performed using the salting out procedure from 1 mL of blood collected in tubes containing acid citrate dextrose from each subject (Miller et al., 1988) or promega genomic DNA isolation kit. Genotyping of this polymorphism at *TLR2 (−196 to −174) del* was performed by allele-specific polymerase chain reaction (PCR). The sequence of forward and reverse primers was adopted from the study by Tahara et al. (2008). The details of sequence of primers are presented in Table 1. The PCR products were observed through electrophoresis on a 3% agarose gel and stained with ethidium bromide for visualization. The observation was finally recorded after assessment of three independent observers. The amplified product of a single band of 286 bp was judged as wild type (insertion/insertion), and a single band of 264 bp was judged as homozygous mutant (deletion/deletion), whereas two bands of 284 and 264 bp were judged as heterozygous (insertion/deletion) for *TLR2 (−196 to −174) del* polymorphism.

**Table 1:** Sequence of primers used for genotyping experiments.

TLR polymorphism	Sequence of primers
*TLR2 (−196 to-174) del* polymorphism	Forward Primer 5′-CACGGAGGCAGCGAGAAA-3′ Reverse Primers 5′-CTGGGCCGTGCAAAGAAG-3
*TLR1 743 A > G* gene polymorphism	Forward Primer 5′-TTGGATGTGTCAGTCAAGACTGTAG-3′ Reverse Primer 5′-GCTTCACGTTTGAAATTGAG-3′ Sensor Probe 5′-TTAAGGTAAGACTTGATAACTTTGG-FL-3′ Anchor probe 5’LCRed640-GTTTGAAGTTTCGCCAGAATACTTAGG

### 2.5 Genotyping for the *TLR1* gene

DNA extraction was performed as described above in section 2.4. Genotyping of *TLR1 743 A > G* gene polymorphism was performed by hybridization probe chemistry in Roche real-time PCR machine. The sequence of *TLR1 gene* (*743 A > G, rs 4833095*), forward primer, reverse primer, sensor probe, and anchor probe was adopted from the study by Sinha et al. (2014). Primers and probes were synthesized from TIB Mol biol, Berlin, Germany. The LightCycler 480 genotyping master mix was procured from Roche Company. The primer and probe sequences used for amplification and the FRET mechanism in detecting *TLR1 743 A > G* gene polymorphism are presented in Table 1. Real-time PCR was performed using 1.5 μL DNA, 2.5 Mm Mgcl2, TLR1 primers at 1.25 pmol, and 250 nm of the *TLR1 743 A > G* sensor probe and anchor probe. The sensor probe was labeled with fluorescein at the 3’end. The anchor probe was labeled with LightCycler red 640 at the 5’end. The amplification was conducted in the LightCycler 480 system (Roche Diagnostics) with the following parameters: initial denaturation at 95°C for 10 min, followed by 40 cycles of amplification with denaturation at 95°C for 10 s, annealing at 60°C for 20 s, and extension at 72°C for 15 s. Subsequently, a melting curve analysis was performed with 1 cycle at 95°C for 1 min, 40°C for 30 s, followed by a temperature increase to 80°C, and final cooling at 40°C.

### 2.6 Stimulation of the peripheral blood mononuclear cells (PBMCs)

Heparin-containing tubes were used to collect eight milliliters of blood samples from eleven PTB, ten MDR-TB, and eleven healthy controls. Density gradient centrifugation was used to isolate PBMCs. One million cells were stimulated with 1 μg/mL of Pam3CSK4 molecule, which is a ligand of TLR2/TLR1 agonist (Hook et al., 2020) of *M.tb* for 24, 48, and 72 h and incubated at 37°C in 5% CO_2_ in a water-jacketed CO_2_ incubator (Thermo Electron Corporation). At each time point, the supernatant was collected and stored at −20°C for cytokine assay.

### 2.7 Measurement of TNF-α, IL-6, and IFN-γ in sera samples and culture supernatant of patients and healthy controls

Cytokines were estimated by sandwich ELISA system using the commercially available reagents from R&D System, United States, following the instructions of the manufacturer. Initially, standardization of PBMC culture was carried out to find out the optimum duration for the production of TNF-α and IL-6 in culture supernatant, which was observed to be highest in 24 h. Cytokines were estimated in culture supernatant of PBMCs and sera samples. The analysis of cytokines in sera samples was performed in 97 PTB, 98 MDR, 45 XDR, and 98 healthy controls.

### 2.8 Statistical analysis

Frequency of genotypes and alleles was counted. Hardy–Weinberg Equilibrium of genotype and allelic frequencies were checked by the chi-square test. Genotype and allele frequencies were compared among the participants by the chi-square test. All statistical analyses were performed by STATA/MP 16.1 software. Comparison of level of cytokine in sera samples and production in culture supernatant was performed by non-parametric analyses (Mann–Whitney test) using GraphPad Prism version 8.01 for Windows (GraphPad software, San Diego, CA) and STATA. The results are depicted in the form of a scattered plots. Comparisons of groups were performed using Kruskal–Wallis equality of proportion rank test. Data were expressed as Mean + _SD or median. *p* < 0.05 was considered as significant.

## 3 Results

### 3.1 Analysis of demographic parameters

A total of 101 PTB, 104 MDR, 48 XDR patients, and 130 healthy controls were included in the study. The mean age of TB patients and healthy controls was found to be significantly different (*p* = 0.007). The male-to-female ratio was similar (*p* = 0.49) among drug-resistant TB patients. On the analysis of various risk factors (smoking and alcoholic habits), among PTB, MDR and XDR cases, no significant difference was observed for smoking (*p* = 0.49) and alcoholic habits (*p* = 0.52), although a significant difference was observed related to consumption (*p* = 0.02) of tobacco among TB patients and healthy controls. A detailed analysis of demographic factors is shown in Supplementary Table S1.

### 3.2 *TLR2 del* polymorphism genotypic analysis

In the present study, genetic polymorphism of the *TLR2* gene was analyzed by allele-specific polymerase chain reaction. The frequencies of genotypes for *TLR2 del* polymorphism did not deviate from Hardy–Weinberg equilibrium in PTB and XDR patients (*p* > 0.05) but were found to have deviated in healthy controls (*p* < 0.05) and MDR cases (*p* < 0.05). The wild-type genotype (Ins/Ins) was observed to be predominant in healthy controls (0.71), PTB (0.73), and MDR-TB participants (0.70), and heterozygous genotypes (Ins/del) were predominant in XDR TB patients (0.57). Table 2 displays the genotype and allele frequency distributions among the different TB patient categories. A pairwise comparison was made between PTB versus MDR, MDR versus XDR, and XDR versus PTB and is presented in Table 3, and TB patients with healthy controls are presented in Table 4. A significant difference was observed in allele and genotypic frequencies of PTB, MDR, and XDR patients for *TLR2 deletion* polymorphism (*p* = 0.0001), and no significant difference was observed in *TLR1 743 A > G* gene polymorphism (Table 2). On analyzing genotype and allele frequencies between MDR-TB and XDR and PTB and XDR-TB, a significant difference was observed between the groups of dominant and over-dominant models (*p* = 0.0001), as shown in Table 3, whereas no significant difference was observed between PTB and MDR-TB with healthy controls (Table 4).

**Table 2:** *TLR2 gene (−196 to −174)* del polymorphism and *TLR1 743 A > G* genotype and allele frequencies among drug resistant TB patients (PTB, MDR and XDR).

*TLR2 (−196 to-174) del* polymorphism	PTB (*N* = 101)	MDR (*N* = 104)	XDR (*N* = 48)	Genotype frequency Chi, *p*, DF	Allele frequency Chi, *p*, DF
I/I	74 (0.73)	73 (0.70)	16 (0.33)	27.58, 0.0001, 4	21.79, 0.0001, 2
I/D	23 (0.23)	22 (0.21)	27 (0.57)		
D/D	4 (0.04)	9 (0.09)	5 (0.10)		
I	171 (0.85)	168 (0.81)	59 (0.61)		
D	31 (0.15)	40 (0.19)	37 (0.38)		
*TLR1 743 A > G*					
A/A	29 (0.29)	31 (0.29)	15 (0.31)	0.22, 0.99, 4	0.018, 0.99, 2
A/G	50 (0.49)	49 (0.48)	22 (0.46)		
G/G	22 (0.22)	24 (0.23)	11 (0.23)		
A	108 (0.54)	111 (0.53)	52 (0.54)		
G	94 (0.46)	97 (0.47)	44 (0.46)		

**Table 3:** *TLR2 gene (−196 to −174) del* polymorphism and *TLR1 743 A > G* genotype and allele frequencies among PTB and MDR, MDR and XDR, PTB and XDR.

	PTB (*N* = 101)	MDR (*N* = 104)	XDR (*N* = 48)	Chi, (DF) & *p* value			OR (95% CI)		
				PTB vs. MDR	MDR vs. XDR	PTB vs. XDR	PTB vs. MDR	MDR vs. XDR	PTB vs. XDR
*TLR2 (−196 to −174) del* polymorphism									
I/I	74 (0.73)	73 (0.70)	16 (0.33)	1.90, (2) & 0.38	20.27 (2) & 0.0001	21.70 (2) & 0.0001			
I/D	23 (0.23)	22 (0.21)	27 (0.57)						
D/D	4 (0.04)	9 (0.09)	5 (0.10)						
I	171 (0.85)	168 (0.81)	59 (0.61)	1.07 (1) & 0.29	12.95 (1) & 0.0001	19.87 (1) & 0.0001	1.31 (0.78–2.19)	2.63 (1.54–4.49)	3.45 (1.97–6.04)
D	31 (0.15)	40 (0.19)	37 (0.38)						
Dominant									
II	74 (0.73)	73 (0.70)	16 (0.34)	0.23 (1) & 0.62	18.38 (1) & 0.0001	21.69 (1) & 0.0001	1.16 (0.63–2.13)	4.70 (2.27–9.74)	5.48 (2.61–11.47)
ID+DD	27 (0.27)	31 (0.29)	32 (0.66)						
Over-dominant									
II + DD	78 (0.77)	82 (0.78)	21 (0.44)	0.07 (1) & 0.78	18.51 (1) & 0.0001	16.35 (1) & 0.0001	0.90 (0.47–1.75)	4.79 (2.29–9.99)	4.36 (2.09–9.05)
ID	23 (0.23)	22 (0.21)	27 (0.56)						
Recessive									
ID+II	97 (0.96)	95 (0.91)	43 (0.90)	1.90 (1) & 0.168	0.122 & 0.72	2.38 (1) & 0.12	2.29 (0.72–7.26)	1.22 (0.40–3.72)	2.18 (0.77–10.2)
DD	4 (0.04)	9 (0.09)	5 (0.10)						
*TLR1 743 A > G*									
A/A	29 (0.29)	31 (0.29)	15 (0.31)	0.11 (2) & 0.94	0.034 (2) & 0.98	0.18 (2) & 0.91			
A/G	50 (0.49)	49 (0.48)	22 (0.46)						
G/G	22 (0.22)	24 (0.23)	11 (0.23)						
A	108 (0.54)	111 (0.53)	52 (0.54)	0.0004 (1) & 0.98	0.017 (1) & 0.89	0.01 (1) & 0.91	1.00 (0.68–1.47)	0.96 (0.59–1.57)	0.97 (0.59–1.5 8)
G	94 (0.46)	97 (0.47)	44 (0.46)						
Dominant									
AA	29 (0.28)	31 (0.30)	15 (0.32)	0.02 (1) & 0.86	0.032 & 0.85	0.106 (1) & 0.75	0.94 (0.52–1.75)	0.93 (0.44–1.94)	0.88 (0.42–1.85)
AG + GG	72 (0.72)	73 (0.70)	33 (0.68)						
Over dominant									
AA+GG	51 (0.51)	55 (0.52)	26 (0.54)	0.11 (1) & 0.73	0.021 (1) & 0.88	0.17 (1) & 0.67	0.90 (0.52–1.56)	0.94 (0.48–1.87)	0.86 (0.43–1.71)
AG	50 (0.49)	49 (0.48)	22 (0.46)						
Recessive									
AG + AA	79 (0.78)	80 (0.77)	37 (0.77)	0.04 (1) & 0.82	0.0005 (1) & 0.98	0.02 (1) & 0.87	1.07 (0.56–2.06)	0.99 (0.44–2.21)	1.06 (0.47–2.40)
GG	22 (0.22)	24 (0.23)	11 (0.23)						

**Table 4:** *TLR2 gene (−196 to −174) del* polymorphism and *TLR1 743 A> G* genotype and allele frequencies between healthy controls and PTB, healthy controls and MDR, and healthy controls and XDR patients.

	Healthy (*N* = 130)	PTB (*N* = 101)	MDR (*N* = 104)	XDR (*N* = 48)	Chi (DF) & *p* value			OR (95% CI)		
					Healthy vs. PTB	Healthy vs. MDR	Healthy vs. XDR	Healthy vs. PTB	Healthy vs. MDR	Healthy vs. XDR
*TLR2 (−196 to −174) del* polymorphism										
I/I	92 (0.71)	74 (0.73)	73 (0.70)	16 (0.33)	1.39 (2) & 0.49	0.07 (2) & 0.96	22.07 (2) & 0.0001	0.88 (0.49–1.57)	1.02 (0.58–1.80)	4.84 (2.39–9.77)
I/D	28 (0.21)	23 (0.23)	22 (0.21)	27 (0.57)						
D/D	10 (0.08)	4 (0.04)	9 (0.09)	5 (0.10)						
I	212 (0.82)	171 (0.85)	168 (0.81)	59 (0.61)	0.77 (1) & 0.37	0.04 (1) & 0.83	15.55 (1) & 0.0001	0.80 (0.48–1.30)	1.05 (0.66–1.67)	2.76 (1.65–4.63)
D	48 (0.18)	31 (0.15)	40 (0.19)	37 (0.38)						
Dominant										
II	92 (0.71)	74 (0.73)	73 (0.70)	16 (0.34)	0.17 (1) & 0.67	0.009 (1) & 0.92	20.59 (1) & 0.0001	0.88 (0.49–1.57)	1.02 (0.58–1.80)	4.84 (2.39–9.77)
ID+DD	38 (0.29)	27 (0.27)	31 (0.29)	32 (0.66)						
Over-dominant										
II + DD	102 (0.78)	78 (0.77)	82 (0.78)	21 (0.44)	0.05 (1) & 0.82	0.005 (1) & 0.94	19.78 (1) & 0.0001	1.04 (0.57–1.99)	0.97 (0.52–1.82)	4.68 (2.32–9.45)
ID	28 (0.22)	23 (0.23)	22 (0.21)	27 (0.56)						
Recessive										
ID+II	120 (0.92)	97 (0.96)	95 (0.91)	43 (0.89)	1.39 (1) & 0.23	0.07 (1) & 0.78	0.33 (1) & 0.56	0.49 (0.15–1.54)	1.13 (0.45–2.83)	1.39 (0.47–4.14)
DD	10 (0.08)	4 (0.04)	9 (0.09)	5 (0.10)						
*TLR1 743 A > G*										
A/A	20 (0.15)	29 (0.29)	31 (0.29)	15 (0.31)	6.133 (2) & 0.047	7.05 (2) & 0.007	5.58 (2) & 0.06	0.45 (0.23–0.85)	0.42 (0.22–0.80)	0.4 (0.18–0.85)
A/G	74 (0.57)	50 (0.49)	49 (0.48)	22 (0.46)						
G/G	36 (0.28)	22 (0.22)	24 (0.23)	11 (0.23)						
A	114 (0.44)	108 (0.54)	111 (0.53)	52 (0.54)	4.21 (1) & 0.04	4.19 (1) & 0.04	3.00 (1) & 0.08	0.67 (0.46–0.98)	0.68 (0.47–0.98)	0.66 (0.41–1.05)
G	146 (0.56)	94 (0.46)	97 (0.47)	44 (0.46)						
Dominant										
AA	20 (0.15)	29 (0.28)	31 (0.30)	15 (0.32)	6.041 (1) & 0.014	7.05 (1) & 0.007	5.58 (1) & 0.018	0.45 (0.23–0.85)	0.42 (0.22–0.80)	0.4 (0.18–0.85)
AG + GG	110 (0.85)	72 (0.72)	73 (0.70)	33 (0.68)						
Over-dominant										
AA+GG	56 (0.44)	51 (0.51)	55 (0.52)	26 (0.54)	1.25 (1) & 0.26	2.22 (1) & 0.135	1.73 (1) & 0.18	0.74 (0.44–1.24)	0.67 (0.40–1.13)	0.64 (0.33–1.23)
AG	74 (0.56)	50 (0.49)	49 (0.48)	22 (0.46)						
Recessive										
AA+AG	94 (0.72)	79 (0.78)	80 (0.77)	37 (0.77)	1.05 (1) & 0.304	0.64 (1) & 0.42	0.41 (1) & 0.52	0.72 (0.39–1.33)	0.78 (0.43–1.41)	0.77 (0.36–1.66)
GG	36 (0.28)	22 (0.22)	24 (0.23)	11 (0.23)						

### 3.3 *TLR1 743 (A > G)* gene polymorphism genotypic analysis

In the present study, *TLR1 743 A > G* allele and genotype frequencies were analyzed by hybridization probe chemistry. *TLR1 743 A > G* gene polymorphism was analyzed in 101 PTB, 104 MDR, and 48 XDR patients and 130 healthy controls. The genotypic and allele frequencies did not deviate from Hardy–Weinberg equilibrium in any of the studied groups. The heterozygous genotype was observed to be predominant in healthy controls (0.57). Furthermore, analyses of genotype frequencies were carried out among the drug-resistant TB patients (PTB, MDR-TB, and XDR-TB). There was no significant difference in frequencies of alleles and genotypes (*p* = 0.99), as shown in Table 2. A significant difference in the frequencies of alleles and genotypes was observed between healthy controls and PTB patients, as well as between healthy controls and MDR-TB patients (*p* = 0.047), (*p* = 0.007) and also in the dominant model (*p* = 0.01), (*p* = 0.007), respectively, as shown in Table 4.

### 3.4 Estimation of TNF-α in relation to severity of tuberculosis disease (PTB, MDR-TB, XDR-TB, and HC) in sera samples and culture supernatant

**Figure 1:**
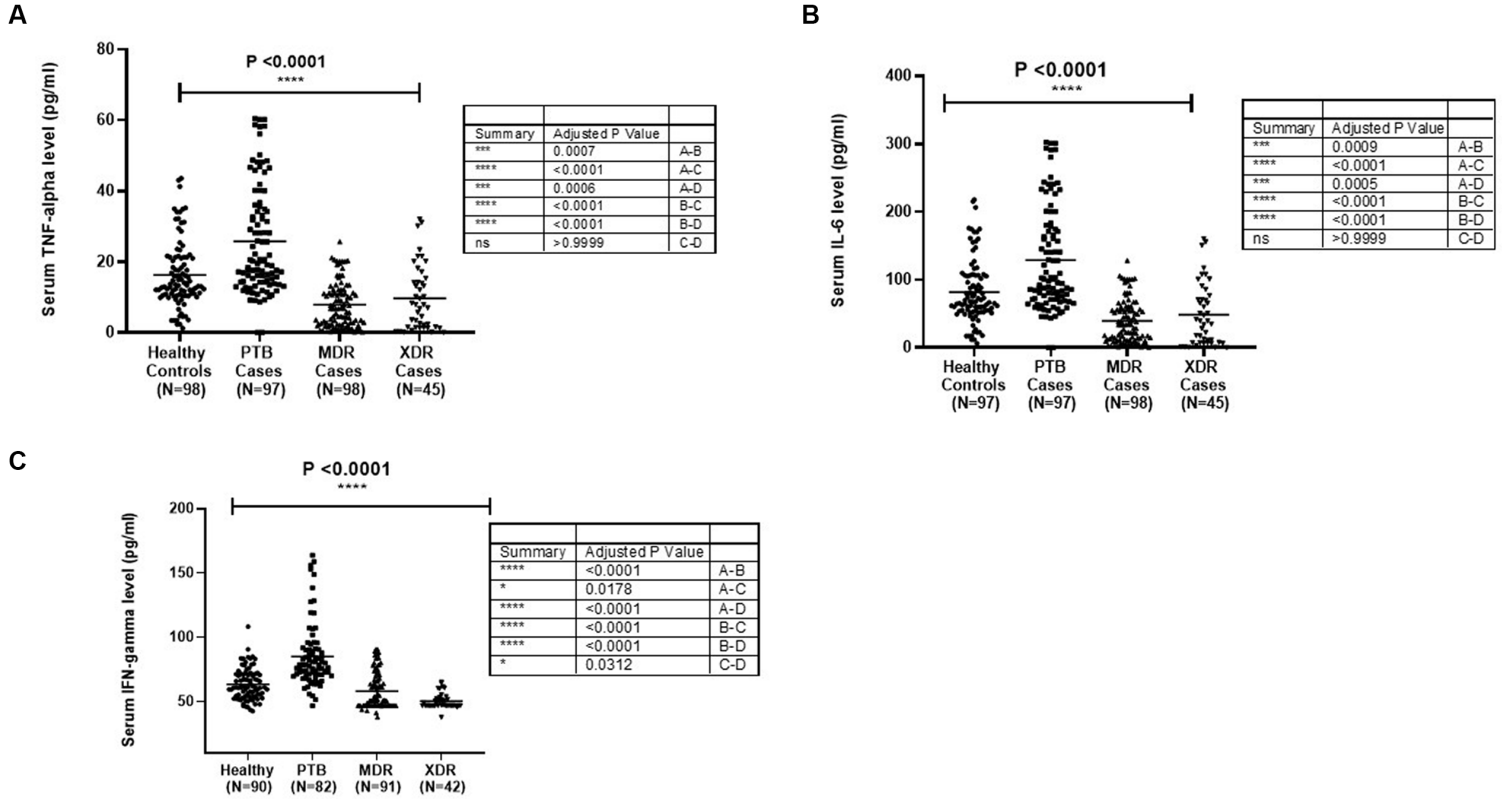
Cytokines [**(A)** TNF-α, **(B)** IL6 and **(C)** IFNγ] level in sera samples of healthy controls PTB, MDR-TB and XDR-TB patients. Each dot represents the level in pg/mL in the serum of one individual. The horizontal bar represents the median level. The level was compared among groups using the Kruskal-Wallis and Mann–Whitney test for pairwise comparison.

The TNF-α level in sera samples among all categories is graphically presented in Figure 1A. The median level of TNF-α in sera samples was observed to be higher in PTB cases (97) (*p* < 0.0001) as compared with healthy controls (98), MDR-TB (98), and XDR cases (45). The level was higher in healthy controls compared with MDR (*p* < 0.0001) and XDR (*p* = 0.0006). There was no significant difference in TNF-α level between MDR and XDR TB patients, as shown in Figure 1A. The participants were stratified as per their TLR2 and TLR1 genotypes, and the level of TNF-α was compared among them. No significant difference was observed in the median level of cytokine among persons having various TLR2 genotypes within healthy controls and all disease types (PTB, MDR, and XDR cases). However, the level of serum TNF-α was higher in healthy controls having Ins/Ins and Ins/Del genotypes compared to XDR TB patients having similar genotypes. The observations are presented in Supplementary Figure S1. When the TNF-α level was analyzed in healthy controls and PTB cases having various TLR1 genotypes, a significant difference was observed in *A/A* (*p* = 0.003), *A/G* genotypes (*p* = 0.011), and *G/G* genotype (*p* = 0.003). There was no difference in the level of TNF-α among three TLR1 genotypes within the group (healthy controls, PTB cases, MDR cases, and XDR cases). The observations are presented in Supplementary Figure S2. The median level of TNF-α in culture supernatant was not significantly different in PTB cases and healthy controls in unstimulated samples as well as in samples stimulated with Pam3CSK4. When the level was compared in MDR and PTB cases, the median level was detected to be higher in MDR cases in unstimulated as well as in stimulated samples. The observations are presented in Supplementary Figure S3.

### 3.5 Estimation of IL-6 with the severity of tuberculosis disease (PTB, MDR-TB, XDR-TB, and HC) in sera samples and culture supernatant

The median level of IL-6 was detected to be higher in PTB cases as compared with healthy controls (*p* < 0.0001) and MDR-TB and XDR-TB cases. The level was higher in healthy controls compared with MDR (*p* < 0.0001) and XDR cases (*p* = 0.0005). The graphical representation of IL-6 level in sera samples is shown in Figure 1B. The participants were stratified as per their *TLR1* and *TLR2* genotypes, and the level of IL-6 was compared among them. No significant difference was observed in the mean level of cytokine among various genotypes within healthy controls and all disease types (PTB, MDR, and XDR cases). However, the level of serum IL-6 was higher in healthy controls having Ins/Ins and Ins/Del genotypes compared to XDR TB patients having similar genotypes. The observations are presented in Supplementary Figure S4. In the *TLR1* gene, a significant difference was observed between healthy controls and PTB cases having *A/A* (0.006) and *A/G* (0.01) genotypes. The observations are presented in Supplementary Figure S5. The median level of IL-6 in culture supernatant was detected to be higher in PTB cases as compared with healthy controls in unstimulated and stimulated (Pam3CSK4) samples. The observations are presented in Supplementary Figure S6.

### 3.6 Estimation of IFN-γ in relation to severity of tuberculosis disease (PTB, MDR-TB, XDR-TB, and HC) in sera samples and culture supernatant

The graphical representation of IFN-γ level in sera samples is shown in Figure 1C. The median level of IFN-γ was detected to be higher in PTB cases as compared with healthy controls (*p* < 0.0001). The level was higher in healthy controls as compared with MDR (*p* = 0.0178) and XDR cases (*p* < 0.0001), as shown in Figure 1C. The participants were stratified as per their *TLR1* and *TLR2* genotypes, and the level of IFN-γ was compared among them. No significant difference was observed in the mean level of IFN-γ level among various genotypes within healthy controls and all disease types (PTB, MDR, and XDR cases). The observations are presented in Supplementary Figure S7. When the IFN-γ level was analyzed in healthy controls and PTB cases having various TLR1 genotypes, significantly higher level was observed in PTB patients *A/A* (*p* = 0.0021), *A/G* genotypes (*p* < 0.0001), and *G/G* genotype (*p* < 0.0001) as compared with healthy controls. No significant difference was observed in the median level of cytokine among various genotypes within healthy controls and all disease types (PTB, MDR, and XDR cases). The observations are presented in Supplementary Figure S8.

## 4 Discussion

TLRs play a crucial role in both innate and adaptive immune responses (Wu et al., 2015). The interaction between TLRs and their corresponding ligands initiates the recruitment of TIR domain-containing adaptor proteins, initiating signaling pathways that protect the host from microbial infections (Ogus et al., 2004). Human TLRs are a family of 10 proteins that selectively recognize microbes and initiate signaling pathways, resulting in the activation of the innate immune response, cytokine production, and the formation of an adaptive immune response (Guan et al., 2010).

The present study addressed the *TLR2 (−196 to −174) del* polymorphism and *TLR1 743 A > G* gene polymorphism among PTB, MDR, XDR, and healthy controls. The frequency of *TLR2 (−196 to −174) ins/del* genotype is observed to be higher among XDR-TB cases (0.57), whereas the frequency of *TLR2 (−196 to −174) ins/del* genotype in MDR-TB and PTB cases is almost equal (0.21 and 0.23, respectively). As a result, a significant difference is observed between XDR-TB and MDR-TB. The allele frequency of *del* allele of *TLR2 (−196 to −174) polymorphism* was observed to be higher among XDR-TB cases (0.38). When the genotypes carrying all *del allele (ins/del + del/del)* were compared against the *ins allele (ins/ins),* a significant difference was observed in both XDR-TB and MDR-TB (*p* = 0.0001) and XDR-TB and PTB (*p* = 0.0001), pointing toward the role of del polymorphism in developing XDR-TB. Velez et al. have reported the most robust association in *TLR2 del* polymorphism with TB in both Caucasian and African populations. This study indicated that *TLR2 del* is the susceptible gene for TB (Velez et al., 2010). According to the report by Khan et al., del polymorphism *TLR2 (−196 to −174)* increased the chance of TB in the Pakistani population (Khan et al., 2014). Tahara et al. identified the effect of this *TLR2 (−196 to −174)* del polymorphism on the severity of *H. pylori*-induced gastritis (Tahara et al., 2008). Noguchi et al. reported that the *ins/ins* and *ins/del* genotypes of this *TLR2 (−196 to −174) del* polymorphism were associated with highly transcriptional activity (Noguchi et al., 2004).

All research studies conducted up to this point has revealed information regarding the susceptibility of a gene polymorphism to either extrapulmonary or pulmonary tuberculosis. *TLR2 (−196 to −174) del* polymorphism may contribute to drug resistance in tuberculosis given that it is associated with disease severity and is found in a higher proportion of drug-resistant cases of the disease. This study represents the first attempt to comprehend the impact of this polymorphism.

The heterozygous genotype (AG) of the *TLR1 743 A > G* polymorphism is predominant in all tuberculosis cases, including PTB (49%), MDR-TB (48%), and XDR-TB cases (46%) and healthy controls (57%). When tuberculosis cases were compared with healthy controls, there was a significant difference found in PTB (*p* = 0.047) and MDR-TB cases (*p* = 0.007). The AG genotype may have a protective role against tuberculosis (TB) since it was more common in healthy controls than in PTB, MDR-TB, or XDR-TB cases.

According to Dittrich et al., the *TLR1 743 A > G* gene is linked to TB protection (Dittrich et al., 2015). According to Salie et al., TLR1 rs 4833095 was found to be significantly associated with tuberculosis diseases (Salie et al., 2015). Moreover, after being stimulated with *M. tuberculosis,* transfected HEK cells carrying the rs 4833095 genotype showed an increased NF-kβ induction (Dittrich et al., 2015). In the meta-analysis conducted by Schurz et al. and Zhou et al., the AG heterozygous genotype of TLR1 rs 4833095 was found to be associated with resistance to tuberculosis in various ethnic groups (Schurz et al., 2015; Zhou and Zhang, 2020). TLR2 plays a significant role in recognizing gram-positive bacteria and other microbial components, such as peptidoglycan and lipopolysaccharides (Akira et al., 2006). TLR1 is a 786 residue type I transmembrane protein having an extracellular domain that is 581 amino acids long and leucine-rich, a transmembrane domain that is 23 amino acids long, and a cytoplasmic toll homology signaling protein that is 181 amino acids long (Zarember and Godowski, 2002). Certain studies have suggested that mutations in the *TLR1* gene, which change the amount of IL-10 and tumor necrosis factor in macrophages, raise the risk of leprosy in Brazil and India (Schuring et al., 2009). The rs 4833095 mutation changes the DNA sequence to replace an adenine with a guanine. It is a missense mutation that causes the asparagine to turn into serine at amino acid position 248 of the protein, lowering TLR1 receptor expression in immune system cells (Uciechowski et al., 2011).

All these studies provide information about the susceptibility of gene polymorphisms with pulmonary TB or extrapulmonary TB. Our observations suggested that a significant difference in frequencies of genotypes among TB patients and heterozygous genotype (Ins/del) of *TLR2 (−196 to −174) del* polymorphism was more prevalent in drug-resistant patients. When the allele was considered, the del allele was predominant in drug-resistant patients (XDR-TB). *TLR2 (−196 to −174) del* polymorphism may have a role in contributing to drug resistance in tuberculosis, though the exact mechanism is still unknown, given its correlation with disease severity and the higher frequency in drug-resistant tuberculosis cases. The present study is the maiden effort to understand the effect of this polymorphism.

We have analyzed various demographic and environmental factors that may contribute to the severity of the disease. A significant difference was observed among various tuberculosis cases related to age, so it was adjusted in all the analyses. No significant difference was observed related to smoking and alcoholism, but a significant difference was observed in relation to tobacco chewing (*p* = 0.02). In the study by Yu et al., they found a positive association of smoking with pulmonary TB (Yu et al., 1988). According to Ghasemian et al., smokers were 2.9 times more susceptible to suffering from pulmonary TB (Ghasemian et al., 2009). The inhalation of smoke activates the alveolar macrophage and produces an inflammatory response, and the longer use of these risk factors diminishes the expression of surface proteins related to antigen presentation by these macrophages (Pankow et al., 1991).

Cytokines play a pivotal role in the host defense mechanism against *M.tb* infection (Anoosheh et al., 2009). Multifunctional cytokines are responsible for the activation, shaping, and response of host body functions. However, a proper balance of cytokines is essential for the right protective responses (Cicchese et al., 2018), since hypo-reactions or hyper-reactions can be harmful and worsen the illness. TNF-α is a proinflammatory cytokine that showed multiple biological effects (Barnes et al., 1993). TNF-α stimulates apoptosis of macrophages, resulting in cell death and subsequent presentation of mycobacterial infection by dendritic cells (Keane et al., 2000). Nakya et al. studied that serum level of TNF-α in pulmonary tuberculosis patients is greater than the control group (Nakaya et al., 1995). The cytokine level is likely to be lower in MDR TB patients (Donnelly et al., 1995). Many investigators have documented that TNF-α level is higher in newly diagnosed TB patients (Juffermans et al., 1998). The result of our study showed that TNF-α level in sera, IL-6, and IFN-γ in PTB patients was greater than the healthy controls, MDR patients, and XDR patients.

Cytokines work in a cascade fashion during TB, where IL-12 controls T1 cytokine (IL-2/IFN-γ) production (Cooper and Khader, 2008; Cooper, 2009; Kumar et al., 2019). The IFN-γ, in turn, activates macrophages (Tötemeyer et al., 2006; Basingnaa et al., 2018) that induce TNF-α secretion for infection containment and mycobacterial growth restriction (Mattos et al., 2010; Nemeth et al., 2011; Basingnaa et al., 2018). TNF-α and IL-1α are the predominant contributors of granuloma formation (Hernandez-Pando et al., 1997; Yao et al., 2017). However, their protective effect was downregulated by the high IL-6/IL-10 levels in association with pSTAT3/SOCS3 expression, which ultimately led to immune suppression and impaired T-cell function (Harling et al., 2019). Moreover, type 1 cytokines (TNF-α and IFN-γ) change their protective nature to promote the spread of disease and use their distinct association with bacterial density to indicate the severity of an infection (Kumar et al., 2019). A significant difference was observed between PTB and MDR cases for TNF-α and healthy and PTB cases for IL-6 cytokine in stimulated samples in culture supernatant samples. It can be deduced that the increased cytokine levels in cases (TNF-α, IL-6 and IFN-γ) by *M. tuberculosis* components are the effect of the defense mechanism mediated through *TLR2 del* and *TLR1 743 A > G* gene polymorphism.

There was a significant difference observed between healthy controls and PTB cases having A/G genotypes for *TLR1 743 A > G* gene polymorphism and healthy controls and XDR cases having Ins/del genotypes for *TLR2 del* polymorphism. So it could be suggested that TLR genotypes may be just one of several factors controlling the cytokine level as TLR2 signaling is known to regulate the activation of various transcription factors (Grassin-Delyle et al., 2020) which in turn regulates the cytokine levels in sera. A variety of factors, in addition to specific genotypes or mutations, may modulate host responses in a way that affects the host’s ability to fight off the continuously multiplying drug-resistant tuberculosis bacteria by up-regulating or down-regulating immunity-related genes and pathways (Ben-Kahla and Sahal, 2016). TLRs, which are a component of the immune system’s physiological barrier, are responsible for identifying mycobacteria and determining which ligands, such as PAM, are linked to them. Any mutation in the TLR gene would prevent it from recognizing the ligand and, as a result, prevent the signaling mechanism from being triggered (Basu et al., 2012). Altered TLR function might lead to reduced recognition of bacteria, contributing to a host environment that is more permissive to the growth of a resistant *M. tuberculosis* stain. It is the complex interplay between host genetics and the pathogen’s ability to evade the immune system (Vijay, 2018; Sia and Rengarajan, 2019). However, further study is required to establish the essential role of *TLR2 del* and *TLR1 743 A > G* gene polymorphism in the recognition of *M.tb* and induction of TNF-α and other cytokines. This may be the first study to analyze the immune response to drug-resistant tuberculosis patients by analyzing *TLR2 (−196 to −174) del* and *TLR1 743 A > G* gene polymorphism.

## Data availability statement

The original contributions presented in the study are included in the article/Supplementary material, further inquiries can be directed to the corresponding author.

## Ethics statement

The studies involving humans were approved by Institute Human Ethics Committee, ICMR-National JALMA Institute for Leprosy and Other Mycobacterial Diseases, Agra. The studies were conducted in accordance with the local legislation and institutional requirements. The participants provided their written informed consent to participate in this study.

## Author contributions

DV: Formal analysis, Methodology, Writing – original draft, Writing – review & editing, Data curation, Software. SS: Writing – review & editing. KM: Conceptualization, Supervision, Funding acquisition, Formal analysis, Writing – review & editing, Software, Project administration. SK: Writing – review & editing, Investigation, Methodology, Validation. NV: Formal analysis, Writing – review & editing. ES: Writing – review & editing. SKB: Writing – review & editing.
