# Supplementary material for: Toll-like receptor 2 (−196 to −174) del and TLR1 743 A > G gene polymorphism—a possible association with drug-resistant tuberculosis in the north Indian population
Source: Front Microbiol. 2024 Jan 24;14:1305974. doi: 10.3389/fmicb.2023.1305974 (PMC10936010; doi:10.3389/fmicb.2023.1305974)
Supplement: Supplementary file 1 [file Data_Sheet_1.docx]

Fig. S1 - Serum TNF-α in sera samples of Healthy controls (a), PTB (b), MDR (c), XDR (d), Healthy (Ins/Ins) and XDR-TB (Ins/Ins) (e), Healthy (Ins/del) and XDR-TB (Ins/del) (f), Healthy (del/del) and XDR-TB (del/del) (g) participants having various *TLR2 (-196 to -174) Ins/del* genotypes. Each dot represents the TNF-α level in pg/ml in the serum sample of one individual. The horizontal bar represents the median level. The level was compared among groups using the Kruskal-Wallis and Mann-Whitney test for pairwise comparison. The p-value is indicated at the top.

Fig.S2 - Serum TNF-α in sera samples of Healthy controls (a), PTB (b), MDR (c), XDR (d), healthy (A/A) and PTB (A/A) (e), Healthy (A/G) and PTB (A/G) (f), Healthy (G/G) and PTB (G/G) (g) samples of participants for *TLR1 743 A>G* genotype. Each dot represents the TNF-α level in pg/ml in the serum sample of one individual. The horizontal bar represents the median level. The level was compared among groups using the Kruskal-Wallis and Mann-Whitney test for pairwise comparison. The p-value is indicated at the top.

Figs.S3 – TNF-α in culture supernatant of healthy controls (a), PTB (b), MDR (c), Healthy (unstimulated) and PTB (unstimulated) (d), Healthy (PAM) and PTB (PAM) (e), PTB (unstimulated) and MDR (unstimulated) (f), PTB (PAM) and MDR (PAM) (g) samples. Each dot represents the TNF-α level in pg/ml in the culture supernatant sample of one individual. The horizontal bar represents the median level. The level was compared among groups using the Kruskal-Wallis and Mann-Whitney test for pairwise comparison. The p-value is indicated at the top.

Fig.S4 - Serum IL-6 in sera samples of Healthy controls (a), PTB (b), MDR (c), XDR (d), healthy (Ins/Ins) and XDR-TB (Ins/Ins) (e), healthy (Ins/del) and XDR-TB (Ins/del) (f), healthy (del/del) and XDR-TB (del/del) (g) participants for *TLR2 (-196 to -174) Ins/del* genotype. Each dot represents the IL-6 level in pg/ml in the serum sample of one individual. The horizontal bar represents the median level. The level was compared among groups using the Kruskal-Wallis and Mann-Whitney test for pairwise comparison. The p-value is indicated at the top.

Fig.S5 - Serum IL-6 in sera samples of Healthy controls (a), PTB (b), MDR (c), XDR (d), healthy (A/A) and PTB (A/A) (e), healthy (A/G) and PTB (A/G) (f), Healthy (G/G) and PTB (G/G) (g) (h) samples of participants for *TLR1 743 A>G* genotype. Each dot represents the IL-6 level in pg/ml in the serum sample of one individual. The horizontal bar represents the median level. The level was compared among groups using the Kruskal-Wallis and Mann-Whitney test for pairwise comparison. The p-value is indicated at the top.

Fig.S6 - IL-6 in culture supernatant of healthy controls (a), PTB (b), MDR (c), healthy (unstimulated) and PTB (unstimulated) (d), healthy (PAM) and PTB (PAM) (e), PTB (unstimulated) and MDR (unstimulated) (f), PTB (PAM) and MDR (PAM) (g) samples. Each dot represents the IL-6 level in pg/ml in the culture supernatant sample of one individual. The horizontal bar represents the median level. The level was compared among groups using the Kruskal-Wallis and Mann-Whitney test for pairwise comparison. The p-value is indicated at the top.

Fig.S7 - Serum IFN-γ in sera samples of Healthy controls (a), PTB (b), MDR (c), XDR (d), Healthy (Ins/Ins) and XDR-TB (Ins/Ins) (e), Healthy (Ins/del) and XDR-TB (Ins/del) (f), Healthy (del/del) and XDR-TB (del/del) (g) samples of participants for *TLR2 (-196 to -174) Ins/del* genotype. Each dot represents the IFN-γ level in pg/ml in the serum sample of one individual. The horizontal bar represents the median level. The level was compared among groups using the Kruskal-Wallis and Mann-Whitney test for pairwise comparison. The p-value is indicated at the top.

Fig.S8 - Serum IFN-γ in sera samples of Healthy controls (a), PTB (b), MDR (c), XDR (d), Healthy (A/A) and PTB (A/A) (e), Healthy (A/G) and PTB (A/G) (f), Healthy (G/G) and PTB (G/G) (g), samples of participants for *TLR1 743 A>G* genotype. Each dot represents the IFN-γ level in pg/ml in the serum sample of one individual. The horizontal bar represents the median level. The level was compared among groups using the Kruskal-Wallis and Mann-Whitney test for pairwise comparison. The p-value is indicated at the top.
